# Glucose disturbances in very low birth weight infants nearing term age—results from the prospective LIGHT-study using continuous glucose monitoring

**DOI:** 10.1007/s00431-025-06284-5

**Published:** 2025-06-27

**Authors:** Itay Nilsson Zamir, Elisabeth Stoltz Sjöström, Johannes van den Berg, Estelle Naumburg, Yonas Berhan, Magnus Domellöf

**Affiliations:** 1 Department of Clinical Sciences, Pediatrics, Umeå University, 90187 Umeå, Sweden; 2 Department of Food, Nutrition and Culinary Science, Umeå University, Umeå, Sweden

**Keywords:** Very low birth weight, Hyperglycemia, Hypoglycemia, Continuous glucose monitoring

## Abstract

The purpose of the study is to assess the prevalence of glucose disturbances (dysglycemia) in very low birth weight (VLBW) infants at 36 weeks postmenstrual age (PMA) using a continuous glucose monitoring (CGM) system and to identify possible risk factors for these disturbances. A prospective observational cohort study (VLBW Infants—Glucose and Hormonal Profiles over Time; LIGHT) included 35 VLBW infants admitted to a single tertiary neonatal intensive care unit during 2016–2019. Perinatal data were registered prospectively. CGM registration was performed at 36 weeks PMA for a period of 48 h. Protracted hyperglycemia and hypoglycemia were defined as > 30 min with glucose concentrations > 8 mmol/L or < 2.6 mmol/L, respectively. A total of 19,907 measurements were retrieved and analyzed. Protracted dysglycemia was found in 68.6% of infants, with 51% of infants experiencing hyperglycemia (4.5% of registration time) and 40% experiencing hypoglycemia (1.2% of registration time). Male sex was associated with longer time spent in hyperglycemia. Insulin treatment prior to 36 weeks PMA was associated with longer time spent in hypoglycemia. CGM values correlated strongly with capillary glucose measurements, and no values fell within clinically dangerous ranges.

*Conclusion*: Protracted dysglycemia is common in VLBW infants even at 36 weeks PMA. Identification of risk factors for dysglycemia might help delineate certain patients in need of careful monitoring of glucose concentrations even when nearing term age. CGM is a promising tool for glucose monitoring in the neonatal intensive care unit.

**Table:** 

**What is Known?**
• *Preterm infants are prone to glucose disturbances in the first weeks of life*.
• *There is some evidence suggesting glucose disturbances are common in preterm infants nearing term age*.
• *Glucose disturbances are associated with morbidity and mortality*.
**What is New?**
• *Continuous glucose registration data confirms subclinical glucose disturbances are common in preterm infants nearing term age*.
• *Male sex and insulin treatment prior to 36 weeks postmenstrual age seem to be associated with glucose disturbances at 36 weeks postmenstrual age*.

**Supplementary Information:**

The online version contains supplementary material available at 10.1007/s00431-025-06284-5.

## Introduction

Glucose concentration disturbances (dysglycemia) are common in preterm infants. Very low birth weight infants (VLBW; < 1500 g) have high rates of hyperglycemia (30–80%) during the first week of life [1–3]. Recent studies show glucose instability may be common even in later stages of the admission period [4–7], when routine glucose monitoring is infrequent. There is no established definition for hyperglycemia in preterm infants, and no clear treatment recommendations can be given. Hyperglycemia yet seems to be associated with a multitude of adverse outcomes [8–16], including increased mortality [6, 17]. Hypoglycemia is common in VLBW infants, with an incidence of at least 30% [2], and is associated with multiple adverse outcomes [18–23]. Like hyperglycemia, there is no clear definition of hypoglycemia, and many definitions are used in clinical practice [24].

Evidence regarding dysglycemia prevalence and risk factors in preterm infants nearing term age is lacking. Continuous glucose monitoring (CGM) has recently been shown to be effective in detecting glucose disturbances otherwise undetected, allowing continuous registration of glucose concentrations over time [2, 25–30]. This study aimed to assess the prevalence of dysglycemia in VLBW infants at 36 weeks postmenstrual age (PMA) using CGM and identify possible risk factors for dysglycemia. We hypothesized that dysglycemia would be common at 36 weeks PMA and that prior dysglycemia during the admission period would be associated with increased prevalence of dysglycemia at 36 weeks PMA.

## Materials and methods

### Study population

This prospective cohort study, the Very Low Birth Weight Infants—Glucose and Hormonal Profiles over Time (LIGHT) study, included 50 VLBW infants born between October 1, 2016, and November 30, 2019, and admitted to the neonatal intensive care unit (NICU) at the University Hospital in Umeå, Sweden. Infants were recruited within the first week after birth. Infants were not included if transported to the NICU later than 24 h after birth or transported from the NICU within the first week of life, did not survive the first week of life, had congenital malformations or chromosomal aberrations, or were registered at county hospitals where the study procedures could not be performed.

Forty-eight infants (96.0%) survived to 36 weeks PMA. Nine infants (18.0%) left the study, and one (2.0%) was discharged before 36 weeks PMA. One infant (2.0%) had an active infection at this age, and it was therefore decided to refrain from applying the CGM sensor. One infant (2.0%) was excluded due to total malfunction of the CGM sensor, and in one infant (2.0%), CGM sensor was not applied due to staff shortage. In total, CGM registrations from 35 (70.0%) infants were included.

### CGM procedure

At 36 ± 1 weeks PMA, a Dexcom G4 sensor (Dexcom Inc., San Diego, CA) was inserted in the frontolateral aspect of the infant’s thigh. Anesthetic cream was applied before sensor insertion. The CGM sensor measures interstitial glucose every 10 s via the glucose oxidase method. An average value is generated every 5 min. The sensor can detect interstitial glucose concentrations from 2.2 to 22.2 mmol/L. The sensor remained in place for a period of 48 h following an initial 2-h warm-up period. This registration period was chosen as it was deemed sufficiently informative for the purposes of the study without risking high drop-out rates. Although the CGM system displayed real-time data, the screen was covered, and the NICU staff were instructed to ignore the readings to prevent bias.

At 36 weeks PMA, the infants have usually already been transferred from the NICU to a unit with a lower level of care in county hospitals. Therefore, the CGM procedure was conducted at five hospitals in Northern Sweden (Sunderbyn, Östersund, Skellefteå, Gällivare, and Umeå University Hospital). Calibrations with point-of-care glucometers (Accu-chek® Inform II, Roche Diagnostics, Basel, Switzerland or HemoCue Glucose 201 +, HemoCue, Ängelholm, Sweden) were done 2 h after sensor insertion and every 12 h thereafter. All infants were exclusively enterally fed during the CGM registration.

Hypoglycemia was defined according to the 2017 recommendations of the Swedish Neonatal Society as a glucose value < 2.6 mmol/L [31]. Protracted hypoglycemia was defined as glucose values < 2.6 mmol/L for at least 30 min without values > 2.8 mmol/L within the interval. Hyperglycemia was defined as a glucose value > 8 mmol/L since this value was previously associated with increased risk for mortality and adverse outcomes [4, 16]. Protracted hyperglycemia was defined as glucose values > 8 mmol/L for at least 30 min without values < 7.6 mmol/L within the interval. Insulin was administered based on the clinical judgment of the attending physician.

### Perinatal data

Perinatal data were prospectively registered according to the study protocol and extracted from medical journals and from the Swedish Neonatal Quality register. Variables included glucose concentrations and data regarding insulin treatment throughout the admission period, gestational age, sex, weight, length, and head circumference measurements at birth and their respective *z*-scores, small for gestational age status, multiple gestation, chorioamnionitis, preeclampsia, method of delivery, antenatal and postnatal corticosteroid treatment, Apgar scores at 1, 5, and 10 min after birth, culture-verified sepsis episodes during admission, inotrope treatment, and duration of mechanical ventilation treatment. Furthermore, data regarding perinatal morbidities including necrotizing enterocolitis, patent ductus arteriosus, intraventricular hemorrhage and its grade, periventricular leukomalacia, retinopathy of prematurity and its stage, and bronchopulmonary dysplasia (BPD) were collected. All glucose concentrations are expressed in mmol/L (conversion factor: mmol/L × 18 = mg/dL).

### Statistical analysis

SPSS Statistical software (IBM SPSS Statistics version 29.0 for Windows, Armonk, NY) was used for statistical analysis. Pearson correlation coefficient was used to assess the agreement between capillary and CGM-generated glucose values. A Bland–Altman plot was constructed using the mean of capillary and CGM measurements as well as the difference between the two. CGM values were analyzed using Clarke error grid analysis, with Zone A representing values within 20% of the reference method, Zone B representing values that differ > 20% from the reference method but that would not have led to inappropriate treatment, Zone C representing values that would have led to unnecessary treatment, Zone D indicating a potentially dangerous failure to detect glucose disturbances, and Zone E indicating hypoglycemia treatment given for hyperglycemia and vice versa [32]. Possible risk factors for dysglycemia at 36 weeks PMA were explored by comparing infants with hypoglycemia only, hyperglycemia only, and combined dysglycemia (both hypoglycemia and hyperglycemia) to normoglycemic infants using Student *t*-test, Mann–Whitney *U*-test, and chi-square test, as appropriate. Possible risk factors associated with the amount of time spent in hypoglycemia or hyperglycemia were explored using Mann–Whitney *U*-test and linear regression models. The significance level was set to *P* < 0.05.

## Results

Clinical characteristics of the LIGHT cohort have been described in previous publications [7, 33]. Briefly, the cohort had a mean gestational age (SD) of 27.2 (2.4) weeks, mean birth weight (SD) of 937 (277) g, 22% received postnatal corticosteroid treatment, and 18% received insulin treatment. Clinical characteristics and anthropometric parameters at birth for infants included in this study are presented in Table 1. No significant differences were found between infants included in this study and infants who did not remain in the study regarding the variables presented in Table 1.

**Table 1:** Clinical characteristics of very low birth weight infants in whom continuous glucose monitoring was performed at 36 weeks postmenstrual age

Characteristic	*N* = 35
Perinatal variables	
Gestational age, mean (SD), weeks	27.3 (2.6)
Female, *N* (%)	23 (65.7)
Multiple gestation, *N* (%)	3 (8.6)
Small for gestational age, *N* (%)	8 (22.9)
Antenatal corticosteroid treatment, *N* (%)	33 (94.3)
Chorioamnionitis, *N* (%)	4 (11.4)
Preeclampsia, *N* (%)	8 (22.9)
Cesarean section, *N* (%)	27 (77.1)
Apgar 1 min, mean (SD)	5 (2)
Apgar 5 min, median (IQR)	7 (3)
Apgar 10 min, median (IQR)	8 (3)
Anthropometric variables	
Weight, mean (SD), grams	929 (276)
Weight *z*-score, median (IQR)	− 1.1 (1.71)
Length, mean (SD), cm	34.7 (3.4)
Length *z*-score, mean (SD)	− 1.76 (1.85)
Head circumference, mean (SD), cm	24.4 (2.5)
Head circumference *z*-score, mean (SD)	− 0.97 (1.02)
Neonatal variables	
Duration (in days) of mechanical ventilation treatment during admission period, median (IQR)	2.0 (10.0)
Treatment with systemic corticosteroids during admission period, *N* (%)	7 (20.0)
Inotrope treatment during admission period, *N* (%)	4 (11.4)
Insulin treatment during admission period, *N* (%)	7 (20.0)
Duration (in days) of insulin treatment, mean (SD)	6.6 (3.5)
Culture-verified sepsis, *N* (%)	3 (8.6)
Intraventricular hemorrhage (IVH)	
No IVH, *N* (%)	26 (74.3)
Grade 1–2, *N* (%)	6 (17.2)
Grade 3–4, *N* (%)	3 (8.6)
Necrotizing enterocolitis, *N* (%)	0 (0.0)
Patent ductus arteriosus, *N* (%)	21 (60.0)
Periventricular leukomalacia, *N* (%)	1 (2.9)
Retinopathy of prematurity (ROP)	
No ROP, *N* (%)	23 (65.8)
Stage 1–2, *N* (%)	6 (17.1)
Stage 3–5, *N* (%)	6 (17.1)
Bronchopulmonary dysplasia, *N* (%)	16 (45.7)
Hyperglycemia > 8 mmol/L during admission period, *N* (%)	31 (88.6)
Hypoglycemia < 2.6 mmol/L during admission period, *N* (%)	16 (45.7)

### CGM data

All CGM registrations were performed at 36 ± 1 weeks PMA. No short-term adverse events, including infections, were associated with CGM use. A total of 19,907 glucose measurements were analyzed. Mean registration time ± SD was 47.4 ± 6.9 h. Sensor malfunction was noted in three infants, yielding only 24 h of data, and in two infants the registration time lasted for 3 days.

**Fig. 1:**
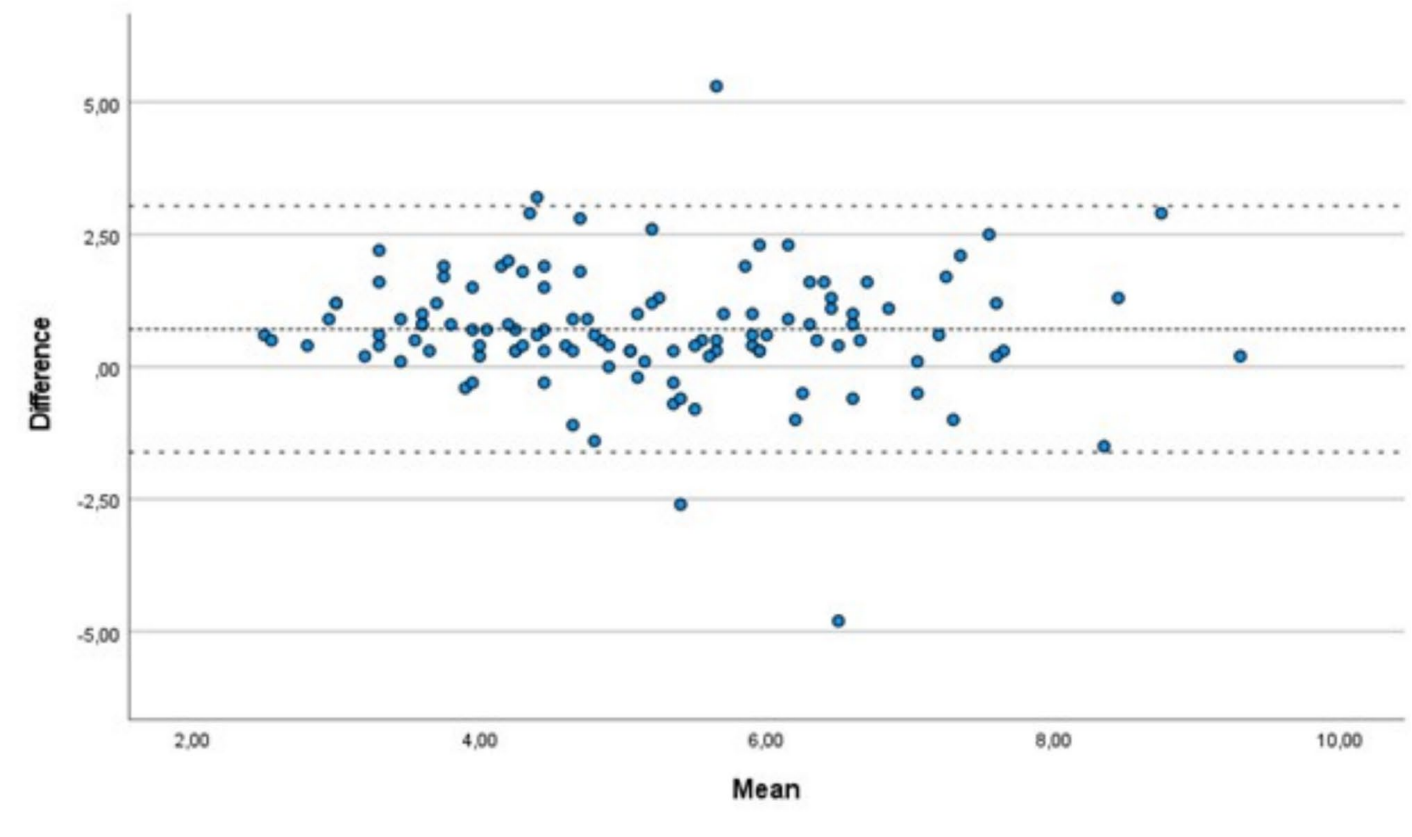
Bland–Altman plot presenting the mean of capillary and continuous glucose monitoring generated glucose values preceding calibration (x-axis) and the difference between values using the two methods (y-axis). In mmol/L

Comparison of the last CGM value before calibration with the respective calibration value (*N* = 118) showed similar distributions (CGM: mean 4.8 ± 1.6 mmol/L; capillary: mean 5.5 ± 1.5 mmol/L). Mean absolute relative difference (MARD; measures the average difference between CGM-generated values and calibration values, a measure of the device’s accuracy) was 18.8%. A strong correlation was found between CGM values and capillary calibration values (*r* = 0.715, *P* < 0.001; online resource 1). Figure 1 shows the Bland–Altman plot for the means and the differences between CGM and capillary measurements. No statistically significant difference was found (*B* − 0.06; *P* = 0.492). Online resource 2 shows the Clarke error grid comparing CGM and capillary measurements. Eighty-three (70.3%) of the CGM values were within Zone A and 35 (29.7%) in Zone B. No CGM values were within Zones C, D, or E.

### Dysglycemia at 36 weeks PMA

Protracted dysglycemia episodes (either hyperglycemia, hypoglycemia, or both) were found in 24 (68.6%) of the 35 infants. Ten infants (28.6%) had only protracted hyperglycemia, six infants (17.1%) had only protracted hypoglycemia, and eight (22.9%) had combined protracted dysglycemia (both hyperglycemia and hypoglycemia). No significant differences were found in the occurrence of protracted glucose disturbances between extremely preterm infants (< 28 gestational weeks) and non-extremely preterm VLBW infants.

Mean percent of time within the ranges 4–8 mmol/L and 2.6–10 mmol/L ± SD was 69.9 ± 12.7% and 97.6 ± 3.5%, respectively. Mean percent of time > 8 mmol/L ± SD was 4.5 ± 6.6%. Mean percent of time < 2.6 mmol/L ± SD was 1.2 ± 2.2%.

Of the 18 infants who had protracted hyperglycemia, three (16.6%) had a single hyperglycemia episode, while nine (50.0%) had 2–5 episodes and six (33.3%) had 6–10 episodes. Of the 14 infants who had protracted hypoglycemia, 6 (42.9%) had only a single hypoglycemia episode, while 8 (57.1%) had 2–5 episodes.

### Risk factors for dysglycemia at 36 weeks PMA

#### Hyperglycemia

Infants who had only protracted hyperglycemia at 36 weeks PMA had significantly shorter birth length (but not birth length *z*-score) and were more likely to have BPD than normoglycemic infants (Online resource 3).

Males, infants with BPD, and infants who have been exposed to hyperglycemia > 8 mmol/L prior to 36 weeks PMA spent significantly longer time in hyperglycemia at 36 weeks PMA (Table 2). Gestational age, weight, length, and head circumference at birth (but not their respective *z*-scores) were significantly negatively associated with the amount of time spent in hyperglycemia at 36 weeks PMA. In a multivariable linear regression model including all the significant factors mentioned above, male sex was significantly associated with longer time spent in hyperglycemia at 36 weeks PMA (*B* 252.172, CI 101.484–402.86, *P* = 0.002).

**Table 2:** Predictors of time spent in hyperglycemia > 8 mmol/L at 36 weeks postmenstrual age. Dichotomous variables followed by continuous variables

Predictor	Median time (IQR) spent in hyperglycemia, min		*P* value
	Males	Females	
Sex	245.0 (500)	0.0 (65)	0.006
	Yes	No	
Multiple gestation	0.0 (45)	50.0 (232.5)	0.184
Cesarean section	0.0 (185)	55.0 (270)	0.602
SGA	0.0 (33.75)	65.0 (240)	0.099
Antenatal corticosteroid treatment	45.0 (197.5)	242.5 (N.A.)	0.706
Amnionitis	105.0 (232.5)	45.0 (185)	0.956
Preeclampsia	35.0 (153.75)	45.0 (280)	0.676
Patent ductus arteriosus	55.0 (225)	0.0 (122.5)	0.343
Intraventricular hemorrhage	70.0 (387.5)	0.0 (198.75)	0.279
Retinopathy of prematurity	60.0 (517.5)	0.0 (175)	0.268
Culture-verified sepsis	0.0 (N.A.)	45.0 (203.75)	0.851
Bronchopulmonary dysplasia	132.5 (287.6)	0.0 (70)	0.045
Systemic corticosteroid treatment	280.0 (375)	0.0 (123.75)	0.054
Hyperglycemia > 8 mmol/L prior to 36 weeks PMA	55.0 (240)	0.0 (0)	0.047
Insulin treatment	210.0 (565)	0.0 (165)	0.115
Hypoglycemia < 2.6 mmol/L prior to 36 weeks PMA	65.0 (240)	0.0 (191.25)	0.415
	B coefficient	CI	
Gestational age (+ 1 week)	− 29.064	− 54.759 to − 3.369	0.028
Birth weight (+ 100 g)	− 28.9	− 52.7 to − 5.2	0.018
Birth weight z-score (+ 1 SD)	0.982	− 46.578 to 48.543	0.967
Birth length (+ 1 cm)	− 25.205	− 44.264 to − 6.146	0.011
Birth length z-score (+ 1 SD)	− 3.24	− 41.729 to 35.248	0.865
Birth head circumference (+ 1 cm)	− 29.64	− 56.665 to − 2.614	0.033
Birth head circumference z-score (+ 1 SD)	10.745	− 59.294 to 80.783	0.757
Apgar score at 1 min (+ 1)	− 1.323	− 29.947 to 27.301	0.926
Apgar score at 5 min (+ 1)	− 16.653	− 51.584 to 18.279	0.339
Apgar score at 10 min (+ 1)	− 30.515	− 75.114 to 14.085	0.173
Duration of mechanical ventilation treatment (+ 1 day)	3.66	− 1.707 to 9.027	0.175

#### Hypoglycemia

Infants who had only protracted hypoglycemia at 36 weeks PMA had significantly lower birth weight, length, and head circumference (but not their respective *z*-scores) and were more likely to have BPD than normoglycemic infants (Online resource 3).

Infants who received insulin treatment prior to 36 weeks PMA spent significantly longer time in hypoglycemia at 36 weeks PMA than infants who did not receive insulin treatment (Table 3). Weight, length, and head circumference at birth (but not their respective *z*-scores) were significantly negatively associated with the amount of time spent in hypoglycemia at 36 weeks PMA. In a multivariable linear regression model including all the significant factors mentioned above, insulin treatment prior to 36 weeks PMA was significantly associated with longer time spent in hypoglycemia at 36 weeks PMA (B 68.607, CI 9.932–127.283, *P* = 0.023).

**Table 3:** Predictors of time spent in hypoglycemia < 2.6 mmol/L at 36 weeks postmenstrual age. Dichotomous variables followed by continuous variables

Predictor	Median time (IQR) spent in hypoglycemia, min		*P* value
	Males	Females	
Sex	0.0 (51.25)	0.0 (45)	0.695
	Yes	No	
Multiple pregnancy	0.0 (35)	0.0 (43.75)	0.682
Cesarean section	0.0 (45)	15.0 (51.25)	0.773
Small for gestational age	0.0 (67.5)	0.0 (40)	0.877
Antenatal corticosteroid treatment	0.0 (42.5)	67.5 (N.A.)	0.521
Amnionitis	0.0 (0)	0.0 (55)	0.101
Preeclampsia	0.0 (0)	0.0 (55)	0.156
Patent ductus arteriosus	0.0 (50)	0.0 (45)	0.458
Intraventricular hemorrhage	0.0 (147.5)	0.0 (41.25)	0.594
Retinopathy of prematurity	15.0 (50)	0.0 (45)	0.530
Culture-verified sepsis	55.0 (N.A.)	0.0 (40)	0.183
Bronchopulmonary dysplasia	30.0 (70)	0.0 (40)	0.082
Systemic corticosteroid treatment	30.0 (70)	0.0 (43.75)	0.149
Hyperglycemia > 8 mmol/L prior to 36 weeks PMA	0.0 (55)	0.0 (0)	0.101
Insulin treatment	55.0 (210)	0.0 (37.5)	0.006
Hypoglycemia < 2.6 mmol/L prior to 36 weeks PMA	0.0 (40)	0.0 (58.75)	0.880
	B coefficient	CI	
Gestational age (+ 1 week)	− 8.019	− 17.005 to 0.966	0.079
Birth weight (+ 100 g)	− 9.8	− 17.9 to − 1.7	0.020
Birth weight ***z***-score (+ 1 SD)	− 3.049	− 19.205 to 13.108	0.704
Birth length (+ 1 cm)	− 7.228	− 13.921 to − 0.536	0.035
Birth length ***z***-score (+ 1 SD)	− 1.458	− 14.557 to 11.641	0.822
Birth head circumference (+ 1 cm)	− 11.913	− 20.836 to − 2.989	0.010
Birth head circumference ***z***-score (+ 1 SD)	− 11.299	− 34.841 to 12.244	0.336
Apgar score at 1 min (+ 1)	− 0.007	− 9.753 to 9.74	0.926
Apgar score at 5 min (+ 1)	− 4.487	− 16.443 to 7.468	0.451
Apgar score at 10 min (+ 1)	− 8.873	− 24.177 to 6.431	0.247
Duration of mechanical ventilation treatment (+ 1 day)	1.373	− 0.443 to 3.189	0.134

#### Combined dysglycemia

Infants who had both protracted hyperglycemia and hypoglycemia at 36 weeks PMA had significantly lower gestational age at birth, lower birth length, weight, and head circumference (but not their respective *z*-scores), were more likely to have BPD, were treated with mechanical ventilation for longer periods, and were more likely to have been treated with insulin during the admission period than normoglycemic infants (Online resource 3).

## Discussion

In this prospective study of 35 VLBW infants, 51% had hyperglycemia and 40% had hypoglycemia during a 48-h CGM registration at 36 ± 1 weeks PMA. Dysglycemia episodes were often recurring. Glucose concentrations were within a narrow target range of 4–8 mmol/L during 70% of the registration time. CGM values strongly correlated with capillary measurements, and no values were within potential risk zones according to Clarke error grid. CGM values differed from capillary glucose values by 18.8% on average. Male sex was associated with longer time spent in hyperglycemia. Insulin treatment prior to 36 weeks PMA was associated with longer time spent in hypoglycemia.

Mola-Schenzle et al. previously presented results from a 72-h CGM registration in 41 VLBW infants who were clinically stable at 32–33 weeks PMA [5]. Baseline characteristics were similar to those in our cohort in terms of gestational age and birth weight. The definitions of hyperglycemia and hypoglycemia in the study by Mola-Schenzle et al. were slightly different (glucose concentrations > 8.3 mmol/L and < 2.5 mmol/L, respectively). Hyperglycemia was detected in 37% of the infants, hypoglycemia in 12% of the infants, and combined dysglycemia in a further 29% of the infants, comparable rates to those reported in our study (29%, 17%, and 23%, respectively). The somewhat lower rates of hyperglycemia and combined dysglycemia and higher rates of hypoglycemia in our study might be explained by older PMA at registration as well as by a developmental mechanism of decreasing insulin resistance [31] and thus decreasing glucose concentrations.

Pertierra-Cortada et al. published data from a cohort study including 60 very preterm infants (< 32 gestational weeks) in whom glucose concentrations were monitored using CGM during 48–72 h around 38 weeks PMA [4]. Hyperglycemia > 7.7 mmol/L was found in 23% of the infants, hypoglycemia < 2.6 mmol/L was found in 10%, and a further 13% had combined dysglycemia. The somewhat higher rates of dysglycemia found in our study can, in a similar manner, be explained by lower PMA in our study compared to the study by Pertierra-Cortada et al.

The high prevalence of subclinical dysglycemia episodes nearing term age, as shown in this study as well as in the studies by Mola-Schenzle et al. and Pertierra-Cortada et al., might call for more rigorous glucose monitoring. On the other hand, this might be a part of a physiological metabolic transition. More recent studies, such as the GLOW study by Harris et al., used CGM to measure glucose concentrations in healthy full-term newborns. It was shown that many of these infants (~ 40%) had glucose concentrations below 2.6 mmol/L and that early term infants had lower mean glucose concentrations than those born at later gestational ages [34]. Previous publications from the LIGHT study have shown that these preterm infants have high levels of insulin resistance markers which decrease towards term age, in parallel with a PMA-dependent decrease in mean glucose concentrations [7, 33].

In the study by Pertierra-Cortada et al., intrauterine growth retardation and female sex were found to be risk factors for hyperglycemia while no risk factors for hypoglycemia could be identified. We present contradicting results in which male sex is a risk factor for hyperglycemia. The reason for this is unclear and should be further explored. Both hyperglycemia and hypoglycemia were associated with smaller birth measurements but not with the respective *z*-scores, implying that it is in fact low gestational age that is the more prominent risk factor. This is supported by the finding that both conditions were associated with increased rates of BPD. Our finding that insulin treatment prior to 36 weeks PMA was significantly associated with longer time spent in hypoglycemia needs confirmation in future studies as well. It is possible that insulin treatment earlier during the admission period affects the infant’s hormonal pathways via early programming, which calls for further evaluation of possible effects of insulin treatment on the hormonal response of the infant.

The strengths of this study include its prospective design and use of CGM, allowing for the registration of many glucose concentrations, enhancing the statistical power. This is one of the few studies using CGM in preterm infants nearing term age. Although studies have shown that more dysglycemia episodes can be detected using CGM than with blood sampling [2, 35], CGM has been shown to have limited point accuracy, especially at the lower ranges of glucose concentrations [2], as well as a drift phenomenon [36], and these devices are not yet recommended for use in patients younger than 2 years of age. Indeed, the CGM system used in this study had a MARD of 19%, which is higher than what is expected of standard CGM systems in use for diabetes (< 10%). This is in line with a meta-analysis that explored the accuracy of multiple CGM devices, where only two studies showed a MARD lower than 10%, with none of the devices satisfying the European Union ISO 15197 requirements [37]. However, using the very same device (Dexcom G4 Platinum) in another study to guide glucose infusion titration has increased the time spent in the euglycemic range [38]. This was confirmed by using another CGM device in the randomized REACT study [39]. Since the time of the LIGHT study, new CGM systems have been developed and were shown to have better accuracy [40]. This emphasizes the large differences between different systems when used in this patient population and the need for further development and careful consideration when using such systems in the NICU setting. Calibrating the CGM system was done using point-of-care glucometers and not by laboratory-analyzed blood samples, which might have affected the accuracy of the calibration. The results presented were not the primary outcome of the LIGHT study, and the sample and group sizes in this study were small, which might have affected its ability to detect significant associations and thus its generalizability. However, results were in line with previously published studies.

In conclusion, more than half of VLBW infants experienced episodes of hyperglycemia at 36 weeks PMA. Hypoglycemia was common as well. This occurred during a period where the infant is believed to have reached a metabolic steady state and glucose concentrations are not monitored frequently. We describe for the first time in this infant population that male sex is associated with longer time spent in hyperglycemia and insulin treatment during the admission period is associated with longer time spent in hypoglycemia nearing term age. It is possible that these infants may require more rigorous monitoring of their glucose concentrations even when nearing term age. Further studies are needed to delineate the nature of these glucose disturbances and their potential consequences. It is also necessary to determine whether the results mirror a developmental metabolic shift towards a more stable metabolic state with decreased insulin resistance at term age.

## Supplementary Information

Below is the link to the electronic supplementary material.

Supplementary file1 (PDF 67 KB)

Supplementary file2 (PDF 122 KB)

Supplementary file3 (PDF 108 KB)

## Data Availability

The data that support the findings of this study are not openly available due to reasons of sensitivity and are available from the corresponding author upon reasonable request. Data are located in controlled access data storage at Umeå University.

## Abbreviations

BPD: Bronchopulmonary dysplasia
CGM: Continuous glucose monitoring
LIGHT: Very low birth weight infants - glucose, and hormonal profiles over time
NICU: Neonatal intensive care unit
PMA: Postmenstrual age
VLBW: Very low birth weight
