# Supplementary material for: Glucose disturbances in very low birth weight infants nearing term age—results from the prospective LIGHT-study using continuous glucose monitoring
Source: Eur J Pediatr. 2025 Jun 27;184(7):452. doi: 10.1007/s00431-025-06284-5 (PMC12204875; doi:10.1007/s00431-025-06284-5)
Supplement: Supplementary file 1 — Supplementary file1 (PDF 67 KB) [file 431_2025_6284_MOESM1_ESM.pdf]

Glucose disturbances in very low birth weight infants nearing term age – results from the prospective LIGHT-study using continuous glucose monitoring

European Journal of Paediatrics

Itay Nilsson Zamir, MD, PhD1 (ORCID ID: 0000-0001-9086-7991), Elisabeth Stoltz Sjöström, RD, PhD2 (ORCID ID: 0000-0002-4649-0653), Johannes van den Berg, RN, PhD1, Estelle Naumburg, MD, PhD1 (ORCID ID: 0000-0001-6090-494x), Yonas Berhan, MD, PhD1 (ORCID ID: 0000-0003-0444-4875), and Magnus Domellöf, MD, PhD1 (ORCID ID: 0000-0002-0726-7029).

Affiliations:

1 Department of Clinical Sciences, Pediatrics, Umeå University, Umeå, Sweden.

2 Department of Food, Nutrition and Culinary Science, Umeå University, Umeå, Sweden.

Adress correspondence to: Itay Nilsson Zamir, Department of Clinical Sciences, Pediatrics, Umeå University, SE-90187 Umeå, Sweden, itay.zamir@umu.se

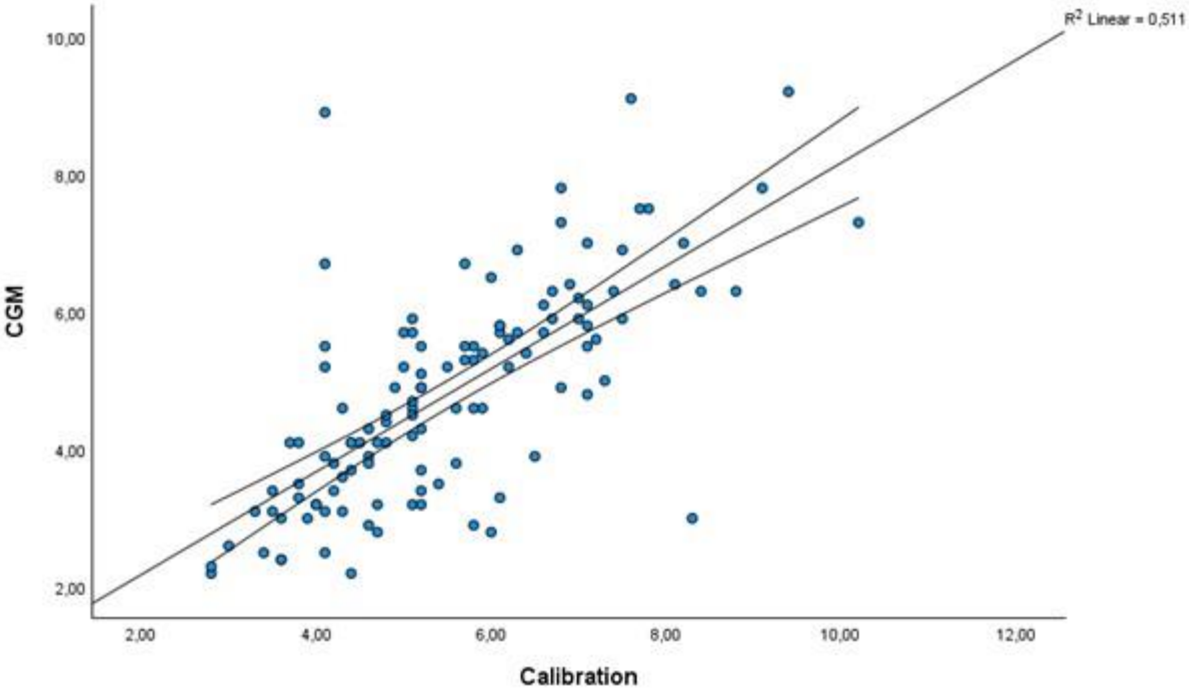

Supplementary figure 1. Correlation and 95% CI between calibration values and continuous glucose monitoring (CGM) generated glucose values preceding calibration. In mmol/L.
