# Supplementary material for: Glucose disturbances in very low birth weight infants nearing term age—results from the prospective LIGHT-study using continuous glucose monitoring
Source: Eur J Pediatr. 2025 Jun 27;184(7):452. doi: 10.1007/s00431-025-06284-5 (PMC12204875; doi:10.1007/s00431-025-06284-5)
Supplement: Supplementary file 2 — Supplementary file2 (PDF 122 KB) [file 431_2025_6284_MOESM2_ESM.pdf]

Glucose disturbances in very low birth weight infants nearing term age – results from the prospective LIGHT-study using continuous glucose monitoring

European Journal of Paediatrics

Itay Nilsson Zamir, MD, PhD1 (ORCID ID: 0000-0001-9086-7991), Elisabeth Stoltz Sjöström, RD, PhD2 (ORCID ID: 0000-0002-4649-0653), Johannes van den Berg, RN, PhD1, Estelle Naumburg, MD, PhD1 (ORCID ID: 0000-0001-6090-494x), Yonas Berhan, MD, PhD1 (ORCID ID: 0000-0003-0444-4875), and Magnus Domellöf, MD, PhD1 (ORCID ID: 0000-0002-0726-7029).

Affiliations:

1 Department of Clinical Sciences, Pediatrics, Umeå University, Umeå, Sweden.

2 Department of Food, Nutrition and Culinary Science, Umeå University, Umeå, Sweden.

Adress correspondence to: Itay Nilsson Zamir, Department of Clinical Sciences, Pediatrics, Umeå University, SE-90187 Umeå, Sweden, itay.zamir@umu.se

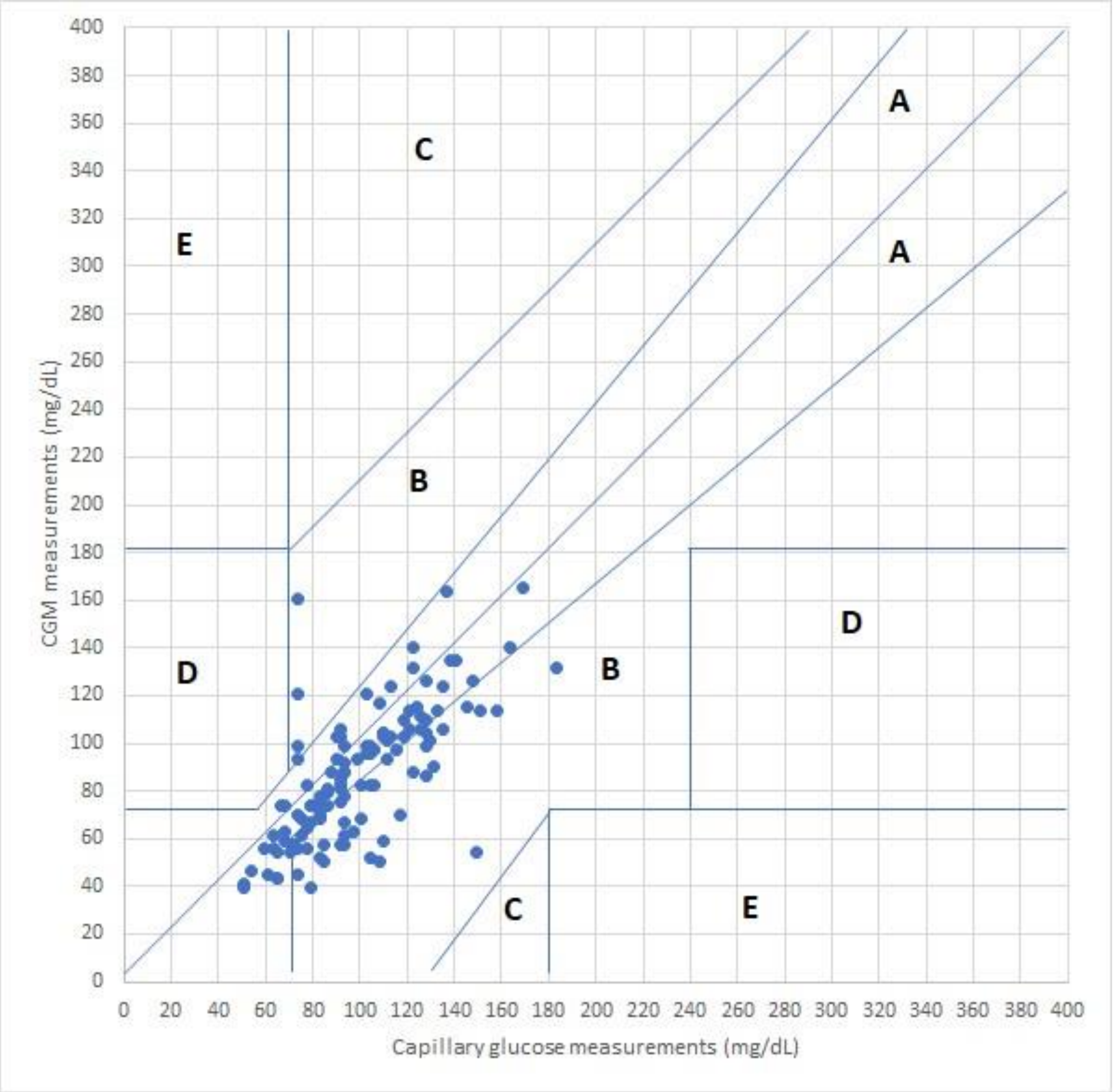

Supplementary figure 2. Clarke error grid comparing capillary glucose measurements (x-axis) with continuous glucose monitoring (CGM) generated glucose measurements (y-axis). In mg/dL. Zone A is defined as values within 20% of the reference method. Zone B includes values that differ >20% of the reference method but that would not lead to inappropriate treatment. Zone C indicates values leading to unnecessary treatment. Zone D indicates potentially dangerous failure to detect glucose disturbances. Zone E indicates hypoglycemia treatment given for hyperglycemia and vice versa.
