# Supplementary material for: Glucose disturbances in very low birth weight infants nearing term age—results from the prospective LIGHT-study using continuous glucose monitoring
Source: Eur J Pediatr. 2025 Jun 27;184(7):452. doi: 10.1007/s00431-025-06284-5 (PMC12204875; doi:10.1007/s00431-025-06284-5)
Supplement: Supplementary file 3 — Supplementary file3 (PDF 108 KB) [file 431_2025_6284_MOESM3_ESM.pdf]

Glucose disturbances in very low birth weight infants nearing term age – results from the prospective LIGHT-study using continuous glucose monitoring

European Journal of Paediatrics

Itay Nilsson Zamir, MD, PhD1 (ORCID ID: 0000-0001-9086-7991), Elisabeth Stoltz Sjöström, RD, PhD2 (ORCID ID: 0000-0002-4649-0653), Johannes van den Berg, RN, PhD1, Estelle Naumburg, MD, PhD1 (ORCID ID: 0000-0001-6090-494x), Yonas Berhan, MD, PhD1 (ORCID ID: 0000-0003-0444-4875), and Magnus Domellöf, MD, PhD1 (ORCID ID: 0000-0002-0726-7029).

Affiliations:

1 Department of Clinical Sciences, Pediatrics, Umeå University, Umeå, Sweden.

2 Department of Food, Nutrition and Culinary Science, Umeå University, Umeå, Sweden.

Adress correspondence to: Itay Nilsson Zamir, Department of Clinical Sciences, Pediatrics, Umeå University, SE-90187 Umeå, Sweden, itay.zamir@umu.se

Supplementary table 1. Predictors of protracted dysglycemia at 36 weeks postmenstrual age (PMA).

Predictor	Glycemia status at 36 weeks PMA			
	Normoglycemia (N=11)	Protracted hyperglycemia only (N=10)	Protracted hypoglycemia only (N=6)	Combined protracted dysglycemia (N=8)
Gestational age, mean ±SD, weeks	28.7 ±2.2	27.3 (2.4)	26.7 (3.3)	25.7 (1.9)**
Birth weight, median (IQR), g	1066 (348)	923 (442)	682 (492)*	703 (417)*
Birth weight z-score, median (IQR), SDS	-0.8 (1.7)	-1.5 (1.6)	-1.8 (2.4)	-0.6 (1.4)
Birth length, mean ±SD, cm	37.1 (2.3)	34.4 (3.1)*	32.8 (3.3)**	33.3 (3.8)*
Birth length z-score, median (IQR), SDS	-1.0 (2.0)	-1.9 (2.8)	-2.5 (2.0)	-0.3 (3.3)
Birth head circumference, mean ±SD, cm	25.9 (1.9)	24.8 (2.2)	23.1 (2.8)*	23.0 (2.3)**
Birth head circumference z-score, mean ±SD, SDS	-1.0 (1.0)	-0.8 (1.1)	-1.4 (1.0)	-0.8 (1.0)
Female, N (%)	8 (72.7)	5 (50.0)	6 (100.0)	4 (50.0)

Predictor	Glycemia status at 36 weeks PMA			
	Normoglycemia (N=11)	Protracted hyperglycemia only (N=10)	Protracted hypoglycemia only (N=6)	Combined protracted dysglycemia (N=8)
Multiple gestation, N (%)	1 (9.1)	1 (10.0)	1 (16.7)	0 (0)
Cesarean section, N (%)	9 (81.8)	8 (80.0)	5 (83.3)	5 (62.5)
Small for gestational age, N (%)	4 (36.4)	1 (10.0)	2 (33.3)	1 (12.5)
Antenatal corticosteroid treatment, N (%)	10 (90.9)	10 (100.0)	6 (100.0)	7 (87.5)
Amnionitis, N (%)	2 (18.2)	2 (20.0)	0 (0)	0 (0)
Preeclampsia, N (%)	3 (27.3)	4 (40.0)	1 (16.7)	0 (0)
Apgar score at 1 min, mean ±SD	5 (2)	6 (3)	6 (3)	5 (3)
Apgar score at 5 min, median (IQR)	7 (2)	7 (3)	8 (2)	7 (2)
Apgar score at 10 min, median (IQR)	9 (3)	9 (3)	8 (3)	8 (2)
Patent ductus arteriosus, N (%)	5 (45.5)	6 (60.0)	4 (66.7)	6 (75.0)
Intraventricular hemorrhage (IVH)				
No IVH, N (%)	10 (90.9)	6 (60.0)	4 (66.7)	6 (75.0)
Grade I-II, N (%)	1 (9.1)	3 (30.0)	0 (0)	2 (20.0)
Grade III-IV, N (%)	0 (0)	1 (10.0)	2 (33.3)	0 (0)
Retinopathy of prematurity (ROP)				
No ROP, N (%)	9 (81.8)	6 (60.0)	3 (50.0)	5 (62.5)
Stage 1-2, N (%)	0 (0.0)	3 (30.0)	1 (16.7)	2 (25.0)
Stage 3-5, N (%)	2 (18.2)	1 (10.0)	2 (33.3)	1 (12.5)
Culture-verified sepsis, N (%)	1 (9.1)	0 (0)	1 (16.7)	1 (12.5)
Bronchopulmonary dysplasia, N (%)	1 (9.1)	6 (60.0)*	4 (66.7)*	5 (62.5)*
Duration (in days) of mechanical ventilation treatment during admission period, median (IQR)	0 (3)	4 (12)	6 (22.5)	3 (27)*
Systemic corticosteroid treatment, N (%)	0 (0.0)	2 (20.0)	2 (33.3)	3 (37.5)

Predictor	Glycemia status at 36 weeks PMA			
	Normoglycemia (N=11)	Protracted hyperglycemia only (N=10)	Protracted hypoglycemia only (N=6)	Combined protracted dysglycemia (N=8)
Hyperglycemia >8 mmol/L during admission, N (%)	7 (63.6)	10 (100.0)	6 (100.0)	8 (100.0)
Insulin treatment during admission, N (%)	0 (0)	1 (10.0)	2 (33.3)	4 (50.0)*
Hypoglycemia <2.6 mmol/L during admission, N (%)	3 (27.3)	7 (70.0)	3 (50.0)	3 (37.5)

Compared with normoglycemic infants at 36 weeks PMA: * P <0.05; ** P <0.01.
